# Man With Post-Traumatic Leg Swelling: A Morel-Lavallée Lesion

**DOI:** 10.7759/cureus.8537

**Published:** 2020-06-09

**Authors:** Emerald Raney, Daniel Ng

**Affiliations:** 1 Emergency Department, Hospital Corporation of America Healthcare, Riverside Community Hospital/University of California Riverside School of Medicine, Riverside, USA; 2 Emergency Department, Hospital Corporation of America Healthcare, Riverside Community Hospital/University California Riverside, Riverside, USA

**Keywords:** morel-lavallee, closed degloving, swelling, post-traumatic

## Abstract

Morel-Lavallée lesions are closed degloving soft tissue injuries that should be considered in post-traumatic patients with persistent pain or swelling. Physicians must keep a high index of suspicion for this commonly missed entity, as early diagnosis is critical to preventing complications. We describe a case of a 46-year-old male who presented to the emergency department following a motorcycle accident. Initial radiographic imaging did not show evidence of osseous injury, and he was discharged home. He later returned with worsening right thigh pain and swelling. Further imaging showed a large fluid collection between the muscle and subcutaneous tissues consistent with a Morel-Lavallée lesion. This case report discusses the common presentation, diagnostic modalities, and treatments to help improve the identification and management of Morel-Lavallée lesions.

## Introduction

Morel-Lavallée lesions are post-traumatic closed degloving soft tissue injuries first described in 1863 by French physician Victor-Auguste-François Morel-Lavallée. The lesion is created by shearing forces that disrupt vessels between subcutaneous tissues and fascia superficial to the muscle [1-4]. A potential space is then created between the tissue and fascia, which then fills with blood, lymph, and necrotic fatty debris. The most common etiologies are motor vehicle accidents, falls, or contact sports [2]. They are most commonly seen in the proximal thigh and trochanteric regions. Other risk factors include body mass index greater than 25 and female gender [2].

## Case presentation

**Figure 1:**
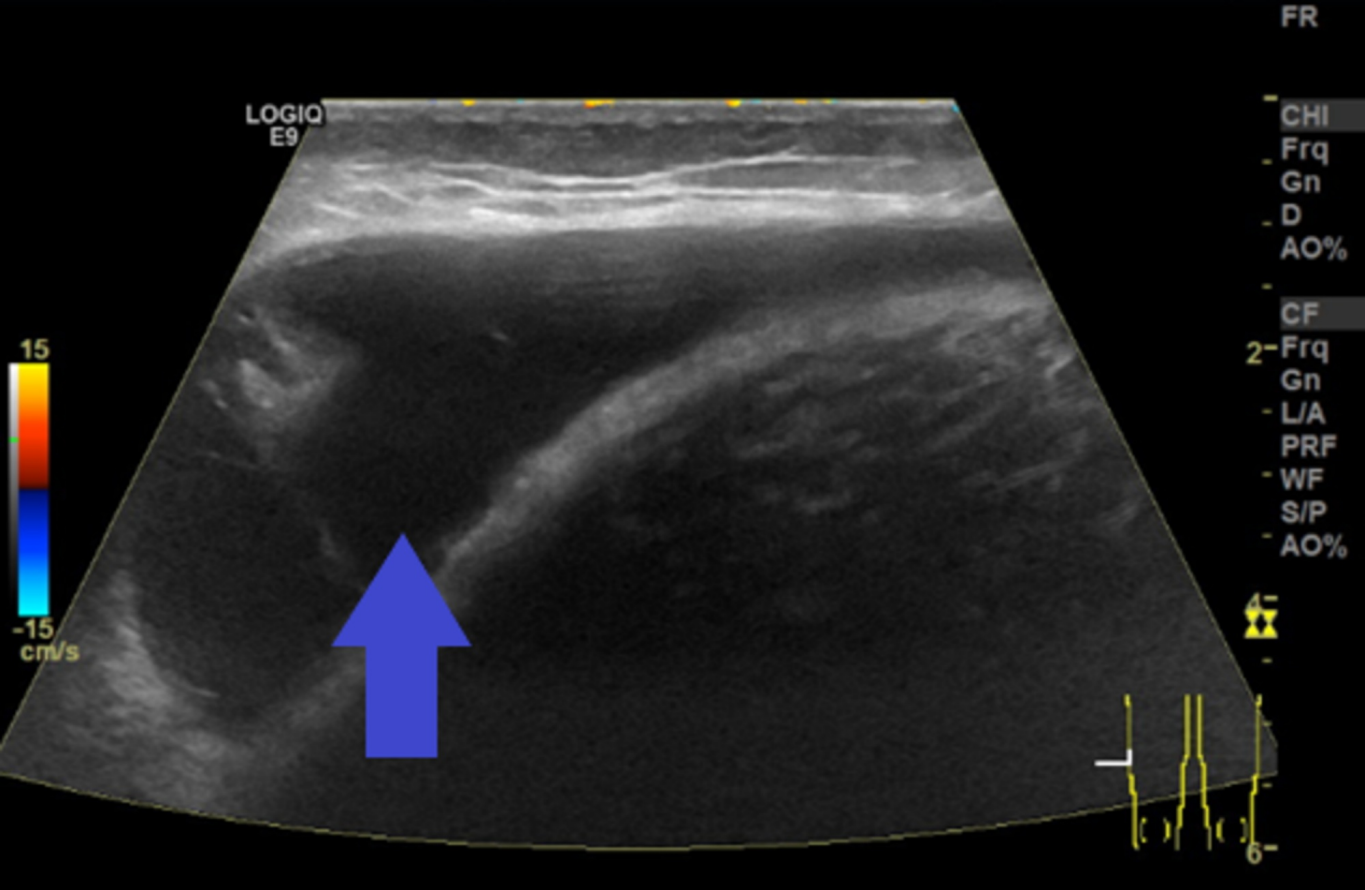
Ultrasound of right lateral thigh showing fluid collection

A 46-year-old male with no past medical history presented to the emergency department for evaluation following a motorcycle accident. He was found to have extensive road rash over the bilateral upper and lower extremities; however, plain radiographs did not demonstrate evidence of osseous injury, and he was discharged home. He returned to the emergency department the next day with worsening right thigh pain and noticed an area of fluctuance. He was able to ambulate but had worsening pain with movement of his leg. Physical exam showed fluctuance on the right lateral thigh, but no bruising and compartments were soft. Bedside ultrasound showed a deep fluid collection. He was diagnosed with a hematoma and advised to follow up with an outpatient MRI. The patient returned to the emergency department 23 days after the initial visit with persistent right-sided thigh swelling, numbness, and paresthesia over the original site of the fluid collection. Formal ultrasound (Figure 1) showed a large fluid collection between the muscle and subcutaneous tissues of the lateral aspect right thigh measuring 14.6 × 7.3 × 2.5 cm with minimal thin internal septations.

**Figure 2:**
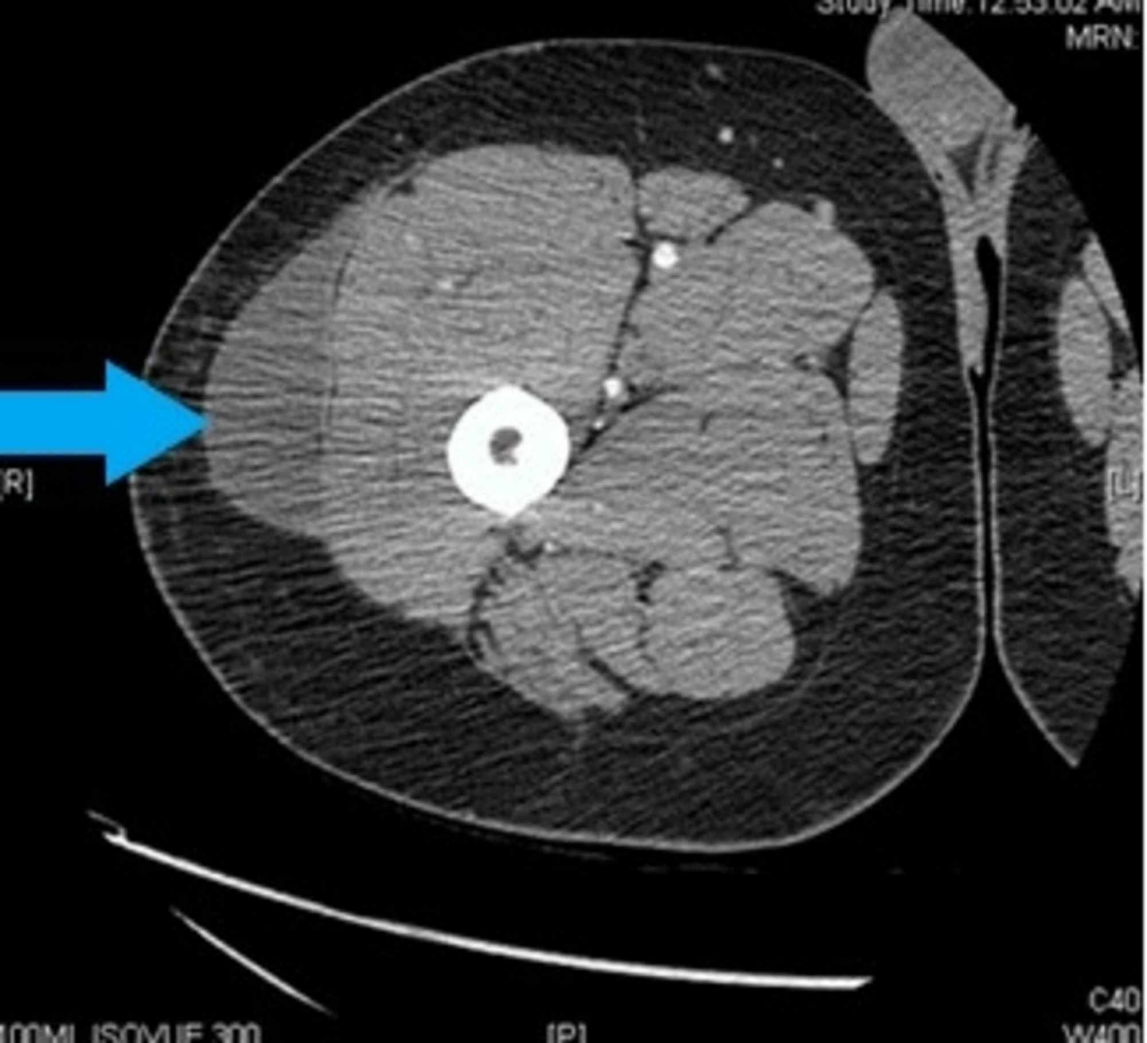
CT with IV contrast right lower extremity (axial view) showing a fluid collection within the deep aspect of the subcutaneous soft tissues

**Figure 3:**
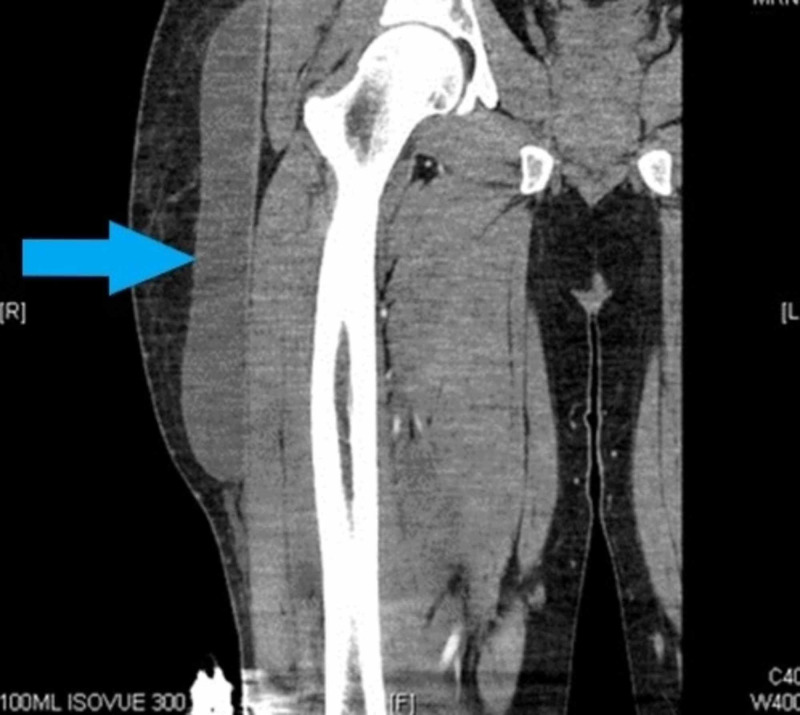
CT with IV contrast right lower extremity (coronal view) with a fluid collection within the deep aspect of the subcutaneous soft tissues

CT scan (Figures 2, 3) showed a fluid collection within the deep aspect of the subcutaneous soft tissues of the lateral right thigh, beginning superficial to the gluteal musculature, extending to the level of the mid/distal right femur, and measuring at least 3 × 7.6 × 23 cm.

These findings, in the setting of recent trauma, were consistent with a Morel-Lavallée lesion. Trauma surgery evacuated the fluid and placed a surgical drain, which was then removed four days later. In follow up with the patient months after the initial injury, he had mild residual paresthesia over the area but no other complications. 

## Discussion

Morel-Lavallée lesions are post-traumatic closed degloving soft tissue injuries. They most commonly occur in the proximal thigh and trochanteric regions and are often associated with pelvic and acetabular fractures [3,4]. The primary locations of the lesions are reported to occur 30.4% in the greater trochanter/hip, 20.1% in the thigh as in our patient, 18.6% pelvis, 15.7% in the knee, and 6.4% in the gluteal region. They have also been described in other anatomic areas, including the abdomen, calf, and head [4].

In the setting of trauma, shearing forces disrupt vessels between subcutaneous tissues and fascia superficial to the muscle [3,4]. Once the vessels are disrupted, the space fills with blood, lymph, and necrotic fat. An inflammatory reaction occurs, which leads to further vessel permeability and leakage, leading to growth of the lesion [2]. Growth of the lesion can be slow and take time to develop, contributing to the delay in diagnosis by weeks to months. The diagnosis is initially missed in a third of cases, especially in polytrauma patients with more obvious injuries [1,4,5]. Over time, the inflammatory reaction can transform granulation tissue into a fibrous peripheral capsule around the fluid-filled space [1,3]. Studies do not discuss a definitive way to identify how long the lesion has been present; however, if a capsule is present, then the lesion is more likely chronic [4]. Patients usually present with soft tissue swelling and fluctuance. Some patients present with pain, firmness, or loss of sensation over the area [4]. There is not an established imaging modality to diagnose the lesion. MRI is preferred, but ultrasound and CT can be used [3]. Mellado and Bencardino have proposed a classification system that describes six lesions based on shape, MRI signal characteristics, enhancement, and presence of a capsule [3,4]. 

Complications can develop if left untreated, including infection of the necrotic material, extensive skin or soft tissue necrosis leading to cosmetic deformities, and like in our patient, paresthesia. Treatment options range from compression dressing, needle aspiration, evacuation through an incision with the placement of a drain, open debridement, or injection of sclerotic agents [2-4]. Management varies based on location, size, severity, and chronicity. Based on current literature, it is unclear if open debridement is superior to percutaneous drainage. Because the vascular supply to the skin has already been compromised, an open debridement could damage the subdermal arterial plexus, the only remaining blood supply. While percutaneous management would help preserve this blood supply, other studies note higher recurrence and infection rates with percutaneous methods [5]. If the capsule remains intact, there is a concern for fluid reaccumulating even after drainage, so for chronic lesions, open debridement with capsule resection may be best [1,4]. Smaller lesions typically have a better response to nonsurgical management versus larger lesions, which likely require surgical intervention. Overall, no single technique has proven significantly more beneficial than others [2,4,5]. 

## Conclusions

Morel-Lavallée lesions are post-traumatic closed degloving soft tissue injuries. They are caused by shearing forces between the superficial and deep fascial layers, thereby giving rise to a hemolymphatic fluid collection. They are commonly missed on initial evaluation and can be slow-growing leading to a delay in diagnosis. If left untreated, this type of injury can lead to long-term complications, including infection and cosmetic deformity. It is important to keep this entity on the differential diagnosis of trauma patients with persistent pain or swelling.
